# A Novel Bioluminescent Biosensor Quantifying Intramolecular Interaction and Levels of Pyroptosis Effector GSDMD

**DOI:** 10.3390/cells13191606

**Published:** 2024-09-25

**Authors:** Tynan Kelly, Simran Bhandari, Madeleine Carew, Rachel Rubino, Christopher Nicol, Xiaolong Yang

**Affiliations:** Department of Pathology and Molecular Medicine, Queen’s University, Kingston, ON K7L 3N6, Canada; 18tnpk@queensu.ca (T.K.); 19sb142@queensu.ca (S.B.); 17mrc7@queensu.ca (M.C.); rubinor@queensu.ca (R.R.); nicolc@queensu.ca (C.N.)

**Keywords:** GSDMD, pyroptosis, programmed cell death, inflammation, NanoBiT, biosensor

## Abstract

Gasdermin D (GSDMD) is a key executor of pyroptosis, a form of inflammation-induced programmed cell death. Recently, GSDMD has been shown to play important roles in the development of various inflammatory-related human diseases including heart failure and cancer, suggesting that it is a promising therapeutic target for these diseases. While extensive studies on GSDMD’s role in pyroptosis have been reported, it is challenging to study its function due to the lack of enzymatic activity of GSDMD. In this study, we used the NanoBiT technology to develop a novel GSDMD bioluminescent biosensor (GSDMD-BS) that detects the amount of non-cleaved GSDMD. This sensor allows us to quantify GSDMD’s intramolecular interactions, the amounts of uncleaved GSDMD after caspase-1 cleavage, and expression levels in living cells. In vitro experiments using purified GSDMD-BS also confirmed the sensor’s accuracy in reporting GSDMD levels and cleavage. Moreover, the potential for in vivo application was demonstrated in a xenograft mouse model. In conclusion, we have developed a GSDMD biosensor that is a valuable tool for real-time monitoring of GSDMD dynamics and pyroptosis. This biosensor will significantly expedite pyroptosis research and can be used for high-throughput screening for drugs targeting GSDMD for the therapy of many inflammation-related diseases.

## 1. Introduction

**Figure 1:**
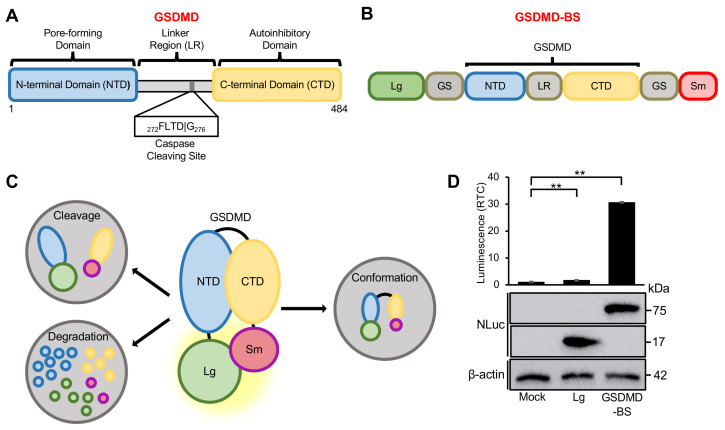
**Schematic of GSDMD domains and pore-forming function.** (**A**) GSDMD contains a pore-forming NTD and an autoinhibitory repressor CTD. Connecting the two is a linker region containing a caspase cleavage site, required for caspase-mediated recognition and proteolytic cleavage. (**B**) Construct designs of the GSDMD intramolecular biosensor. The GSDMD-BS was engineered by attaching the NanoBiT components, LgBiT and SmBiT, to the N- and C-termini of full-length GSDMD, respectively. Each NanoBiT component is connected to GSDMD via a flexible glycine–serine linker region (GS). (**C**) Schematic demonstration of reduced GSDMD-BS bioluminescent signal intensity after degradation, cleavage, or conformational changes in GSDMD. (**D**) Validation of GSDMD-BS by luciferase assays. Upper panel: Luciferase assay of GSDMD-BS. The biosensor exhibited a significant increase in luminescent activity compared to cells transfected with an empty plasmid or LgBiT alone (30-fold, **, *p* < 0.01), demonstrating that luciferase complementation occurs within GSDMD-BS. LgBiT alone does not produce a significant change in background signal. Henceforth, luminescent activity is normalized to control signal (mean ± SD; *n* = 3). Lower panel: GSDMD-BS was detectable by Western blot using anti-NLuc antibody (specific to LgBiT). β-actin was used as an internal control for all Western blots.

Gasdermin D (GSDMD) is the primary executor of pyroptosis, a lytic and inflammatory form of cell death [1,2,3,4,5,6]. Structural analysis shows that GSDMD is composed of a pore-forming N-terminal domain (NTD) and an autoinhibitory C-terminal domain (CTD), interspaced with a linker region containing a caspase cleavage site (^272^FLTD^278^) (Figure 1A) [7]. In its closed form, GSDMD’s NTD interacts with its CTD. When activated by specific inflammatory signals like pathogen infection or cellular stress, GSDMD is cleaved at its cleavage site by inflammatory caspases 1/4/5/11 [7,8]. The cleaved GSDMD NTD oligomerizes, resulting in pore formation on the cell membrane and subsequent pyroptosis with ruptured cells releasing inflammatory contents like cytokines [8,9]. GSDMD-induced pyroptosis plays a critical role in host defense and immune surveillance, as well as in conditions like sepsis, Alzheimer’s Disease, heart failure, and cancer [1,2,3,10,11,12,13]. Recent studies have further emphasized the diverse roles of GSDMD in both normal physiology and disease states. Chi et al. (2024) demonstrated that GSDMD-mediated metabolic crosstalk promotes tissue repair, highlighting its importance beyond inflammatory responses [14]. Additionally, Fontana et al. (2024) showed that small-molecule GSDMD agonism in tumors can stimulate antitumor immunity without systemic toxicity, opening new avenues for cancer immunotherapy [15]. These findings underscore the need for tools that can accurately monitor GSDMD activity in various contexts. While extensive studies have been carried out on the role of GSDMD in pyroptosis [1,2,8,16], except for semi-quantitative Western blot analysis of GSDMD in vitro, there is no sensitive method to accurately monitor and quantify GSDMD dynamics including the changes in GSDMD’s conformation, levels (e.g., degradation), and caspase-induced cleavage in living cells during pyroptosis or under post-translational modification. The development of this method to quantify GSDMD dynamics is critical in evaluating GSDMD’s function in pyroptosis-dependent and pyroptosis-independent physiological and pathological processes [1,2,16,17] and in screening for novel upstream GSDMD regulators and small-molecule drugs targeting GSDMD for disease therapy [1,3,18].

Our research group has previously engineered a variety of luminescent biosensors to quantify the abundance and function of crucial Hippo pathway components. These include sensors for LATS kinase activity, Merlin’s intramolecular interactions and expression levels, and the protein–protein interaction between YAP/TAZ and TEAD. These tools have proven effective both in cellular and animal models [19,20,21,22,23,24,25]. By leveraging these biosensors, we have successfully identified multiple upstream regulators and small-molecule compounds that influence Hippo pathway signaling, opening new avenues for potential cancer therapies. In this study, we aim to construct a GSDMD biosensor using a similar NanoBiT split luciferase system that has been successfully applied in our previous work [20,21,25]. The NanoBiT system is based on NanoLuc luciferase, which is split into two subunits: the 18 kDa LgBiT (Lg) subunit and the 1.3 kDa, 11-amino acid SmBiT (Sm) subunit [24,26,27]. To create the GSDMD biosensor (GSDMD-BS), we fused LgBiT and SmBiT to the N- and C-termini of full-length GSDMD, respectively (Figure 1B).

We hypothesize that in its inactive ‘closed’ conformation, GSDMD’s N-terminal domain (NTD) interacts intramolecularly with its C-terminal domain (CTD), allowing LgBiT and SmBiT to complement each other and form a functional NanoLuc luciferase that emits luminescence in the presence of its substrate, Furimazine (Figure 1C). Conversely, in the ‘open’ conformation, induced by caspase cleavage or post-translational modifications, the NTD separates from the CTD, leading to the dissociation of LgBiT and SmBiT and a corresponding reduction in bioluminescent signal (Figure 1C). A similar reduction in the GSDMD-BS signal can also be observed when GSDMD undergoes degradation. Therefore, this biosensor represents a powerful tool for real-time monitoring of GSDMD’s molecular function and can be applied in future studies to provide unique insights into both the pyroptosis-dependent and -independent roles of GSDMD.

## 2. Materials and Methods

### 2.1. Biosensor Design and Construction

GSDMD is structured with a pore-forming N-terminal domain (NTD) and an autoinhibitory C-terminal domain (CTD), connected by a linker region that includes a caspase cleavage site (^272^FLTD^278^) (Figure 1A). To create the GSDMD biosensor (GSDMD-BS), we designed a construct comprising three main components: the N-terminal LgBiT element, the complete human GSDMD sequence (accession number NM_024736), and the C-terminal SmBiT element. We amplified the human GSDMD gene using PCR techniques. The SmBiT luciferase component was incorporated by designing a reverse PCR primer with a 33-nucleotide SmBiT-encoding overhang, along with a sequence encoding a flexible glycine–serine (G/S) linker to ensure optimal luciferase reconstitution. This design allowed us to attach SmBiT and its G/S linker to the C-terminus of the GSDMD PCR product (Table S1). The resulting GSDMD-SmBiT fusion was then enzymatically digested and inserted into the EcoR1/Nhe1 sites of the pBiT1.1-N vector (Promega, Madison, WI, USA). This vector already contained an N-terminal LgBiT-encoding sequence with a G/S linker, positioned in-frame with our GSDMD-SmBiT insert. The final product was an intramolecular GSDMD biosensor with the following structure: LgBiT–linker–GSDMD–linker–SmBiT (Figure 1B).

### 2.2. Site-Directed Mutagenesis

We employed an overlapping PCR strategy to perform site-directed mutagenesis, following a protocol detailed in a previous publication [28]. The specific primers used for mutagenesis and cloning are listed in Table S1.

### 2.3. Cell Culture

For our experiments, we used HEK293T cells (human embryonic kidney; ATCC, Cat#CRL-3216). These were maintained in Dulbecco’s modified Eagle’s medium (DMEM; D6429; Sigma-Aldrich, Oakville, ON, Canada) supplemented with 10% Fetal Bovine Serum (FBS) and 1% Penicillin/Streptomycin (P/S; Invitrogen, Carlsbad, CA, USA). All cell cultures were kept at 37 °C in a humidified incubator with 5% CO_2_.

### 2.4. Protein Extraction and Western Blot Analysis

We extracted cellular proteins using 1 × Passive Lysis Buffer (1 × PLB; Promega, Madison, WI, USA), adhering to the manufacturer’s protocol. For Western blot analysis, we first blocked the membranes with 5% skim milk solution for 60 min at room temperature. We then probed the membranes with primary antibodies: anti-GSDMD (Abcam #210070, Waltham, MA, USA, 1:1000 dilution), anti-β-actin (Sigma Aldrich #A5441; 1:10,000 dilution), or anti-NanoLuc (NLuc) (Promega#N7000, Madison, WI, USA, 1:1000 dilution). The primary antibody incubation was carried out for 1 h at room temperature or overnight at 4 °C. After washing, we applied HRP-conjugated secondary antibodies at a 1:2500 dilution for 15 min. We then treated the membranes with Clarity™ Western ECL Substrate (Bio-Rad, Hercules, CA, USA) for 1 min to develop the signal. Images were captured using a ChemiDoc™ MP Imaging System (Bio-Rad) and band intensities were quantified using ImageJ software v 1.54.

### 2.5. Lentivirus Production and Stable Cell Line Generation

To establish stable cell lines, we introduced the GSDMD-BS transgene into MCF7 cells using a lentiviral approach. We first inserted the GSDMD-BS sequence into the pWPI lentiviral vector, which also expresses GFP. For lentivirus production, we grew HEK293T cells to 90–100% confluence in 60 mm dishes. We then transfected these cells with a mixture of 1 µg GSDMD-BS/pWPI, 0.75 µg psPAX (for lentiviral packaging), and 0.25 µg PMD2G (for viral envelope) using Polyjet Transient Transfection Reagent as per the manufacturer’s guidelines (SignaGen, Frederick, MD, USA). After 24 h, we added sodium butyrate to the culture medium (final concentration 10 mM) to boost lentivirus production. Following an additional 24 h incubation, we harvested the virus-containing medium, filtered it through a 0.45 μm filter, and aliquoted and flash-froze it in liquid nitrogen. We then infected MCF7 cells with varying amounts of this lentivirus in the presence of 8 µg/mL Polybrene. For subsequent experiments, we selected MCF7-GSDMD-BS cells that showed >80% GFP positivity.

### 2.6. Luciferase Assays

For our luciferase assays, we transfected cells with the GSDMD-BS construct, either alone or in combination with other plasmids, using Polyjet Transfection Reagent (SignaGen). We then lysed the cells with 1 × PLB (Promega, Madison, WI, USA). To measure luciferase activity, we employed the Nano-Glo Live Cell Assay System (Promega) with furimazine as the substrate, following the manufacturer’s protocol as described in our previous work [20]. We quantified luminescent activity using either a Turner Biosystems 20/20 Luminometer or a GloMax Navigator Microplate Luminometer (both from Promega). In all cases, we report luciferase assay data as luminescence relative to a no-biosensor control (RTC). Each transfection was performed in triplicate, and the experiments were repeated at least twice. The mean and standard deviation for each transfection were calculated. The statistical analysis was performed with a Student *t*-test or a one-way ANOVA using Prism program.

### 2.7. Bioluminescent Imaging (BLI) Analysis in Cells In Vitro

We conducted our in vitro BLI analysis by seeding 16 × 10^4^ MCF7 cells or increasing numbers (1–16 × 10^4^) of MCF7-GSDMD-BS cells into each well of a 12-well plate. After adding Nano-Glo Live Cell substrate (Promega, Madison, WI, USA), we measured bioluminescent signals using a Perkin Elmer IVIS Ilumina III imaging system.

### 2.8. Xenograft Mouse Model and In Vivo Imaging

A total of n = 3 immunocompromised female 12-week-old Rag2^−/−^; Il2Rγ^−/−^ mice per experiment were anesthetized with 5% isoflurane for induction and 3% isoflurane to maintain, and injected orthotopically into #4 and #9 mammary fat pads of each mouse, respectively, with either 5 × 10^6^ MCF7 or MCF7-GSDMD-BS cells suspended in 50 µL sterile 1 × PBS containing 0.22 µmol of fluorofurimazine using a Hamilton syringe. Bioluminescent imaging was performed using an IVIS Lumina Series III in vivo imaging system (PerkinElmer) at 5, 10, 15, 30, 45, 60, and 75 min post-injection. Images were acquired with 0.5 s exposure time, and analyzed using Living Image 3.2 software (PerkinElmer, Woodbridge, ON, Canada) and a normalized luminescence count color scale min = 1000; max = 25000. Signal intensity was quantified as total flux (photon/s) in the defined region of interest in the implantation sites. All procedures and protocols for the in vivo xenograft analysis using mice were approved by the Queen’s University Animal Care Committee (Permit# #2021-2181) in accordance with the Canadian Council on Animal Care guidelines, and performed as described previously [20]. This study was funded by the Canadian Cancer Society-Challenge grant (Grant#369904) and Canadian Institute of Health Research (CIHR) grants 186142 and 148629, which adhere to all ethical guidelines for animal research.

### 2.9. Purification of His-Tagged GSDMD-BS

We subcloned the LgBiT-GSDMD-SmBiT construct into a pET28b vector and transformed it into BL21(DE3) competent bacterial cells. We selected a single colony and inoculated it into 25 mL of 2xYT medium containing kanamycin, then incubated this culture overnight at 37 °C with shaking at 250 rpm. The next day, we diluted the overnight culture to an OD600 of 0.2 in 250 mL of 2xYT medium and continued incubation at 37 °C until the OD600 reached between 0.6 and 0.8. We then induced protein expression by adding 0.3 mM IPTG (isopropyl β-D-1-thiogalactopyranoside) and incubating overnight at 25 °C. We harvested and lysed the bacterial cells by sonication, then centrifuged the lysates to collect the soluble fraction. From this, we purified His-tagged proteins using Ni-NTA affinity chromatography. We concentrated the purified proteins using an Amicon Ultra-4 Centrifugal Filter Unit (Millipore-Sigma, Oakville, ON, Canada) in a buffer containing 30 mM Tris-HCl (pH 7.5), 150 mM NaCl, 5 mM MgCl_2_, and 3 mM DTT. Finally, we quantified the concentrated proteins by SDS-PAGE, aliquoted them, and stored them at −80 °C for future use.

### 2.10. Analyzing the Cleavage of Purified GSDMD-BS by Caspase-1 In Vitro

Triplicate samples of 100 ng of purified His-GSDMD-BS fusion protein were incubated with increasing amounts [0–1 Unit (U)] of active caspase-1 (CASP1; Enzo, Farmingdale, NY, USA, Cat#BML-SE168; 100 U/µL) in 1× caspase buffer (50 mM HEPES, pH 7.4, 100 mM NaCl, 0.1% CHAPS, 1 mM EDTA, 10% glycerol, 10 mM DTT) at 30 °C for 30 min. The reaction mixtures were then subjected to luciferase assay and Western blot analysis to assess CASP1 activity and His-GSDMD-BS cleavage, as described in previous sections.

## 3. Results

### 3.1. Development and Validation of GSDMD-BS

The NanoLuc components LgBiT and SmBiT were cloned onto the N and C-terminus, respectively, of full-length GSDMD (Figure 1B). After transfection of GSDMD-BS into HEK293T cells, the GSDMD-BS showed a dramatic (~30-fold) increase in luminescent activity as compared to untransfected (mock) and pBiT1.1-N vector (expressing LgBiT alone)-transfected controls (Figure 1D, upper panel), demonstrating that luciferase complementation is occurring as predicted. Expression of the LgBiT and GSDMD-BS was also verified by Western blot (Figure 1D, low panel).

**Figure 2:**
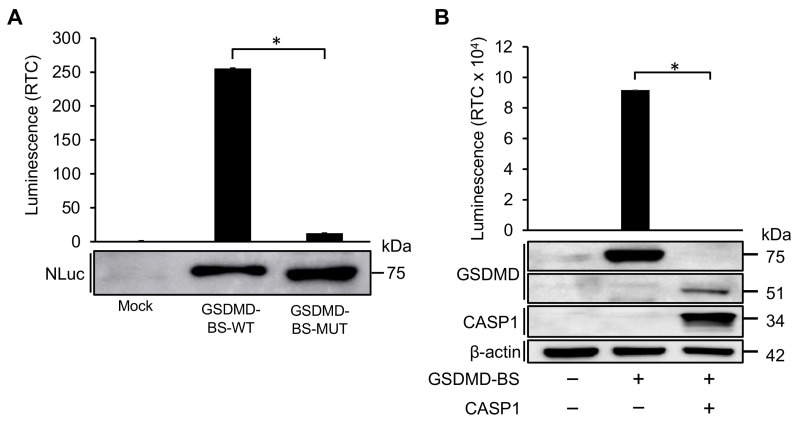
**GSDMD-BS activity is dependent on NTD-CTD intramolecular interaction.** (**A**) Luciferase assay of GSDMD-WT and GSDMD-MUT. Upper panel: Luciferase assays. GSDMD-BS activity was confirmed by introducing several mutations in the CTD responsible for binding the repressing the NTD. GSDMD-MUT had a considerably low signal compared to GSDMD-BS, demonstrating GSDMD-BS signal is attributed to luciferase complementation upon NTD-CTD binding (mean ± SD; *n* = 3). Lower panel: Equivalent amounts of lysates (Mock, GSDMD-WT, GSDMD-MUT) used for luciferase assays (upper panel) were also used for Western blot analysis using anti-Nluc antibody. (**B**) Reduced GSDMD-BS activity after CASP1 cleavage of GSDMD. GSDMD-BS was transfected alone or together with CASP1-FLAG into HEK293T cells, then protein lysate was extracted for luciferase assay and Western blot. Upper panel: Luciferase assays. In the presence of catalytically active CASP1, the GSDMD-BS luminescent signal drops dramatically (~90 000-fold; *, *p* < 0.05) compared to GSDMD-BS alone (mean ± SD; *n* = 2). Lower panel: Western blot analysis of GSDMD-BS and CASP1-FLAG levels by Western blot using anti-GSDMD (detect GSDMD-NTD) and anti-FLAG (detecting CASP1-FLAG). While full-length GSDMD-BS (75 kDa) is present when expressed alone, cleaved GSDMD (51 kDa; cleaved component contains GSDMD-NTD and LgBiT) is only detected when co-transfected with CASP1.

Next, we intended to validate the hypothesis that the luciferase activity for cells transfected with GSDMD-BS is caused by complementation of LgBiT and SmBiT due to GSDMD’s NTD-CTD intramolecular interaction. For this, we first generated a GSDMD mutant (GSDMD-MUT) that contains four mutations (L290A/E293A/Y373A/E293A), which has previously demonstrated to disrupt GSDMD’s NTD-CTD intramolecular interaction [29]. Equal amounts of wild-type (GSDMD-BS-WT) or mutant (GSDMD-BS-MUT) GSDMD-BS plasmid were transfected into HEK293T cells. As expected, at similar levels of protein expression, compared to GSDMD-GS-WT, the luciferase activity is almost abolished in GSDMD-GS-MUT (Figure 2A,B), suggesting that the luciferase signal is indeed caused by complementation of LgBiT and SmBiT after the intramolecular interaction of the NTD and CTD of GSDMD. In addition, we further tested whether this biosensor could monitor GSMD cleavage by caspase as we anticipated (Figure 1C, lower panel). Significantly, co-transfection of plasmid expressing active caspase-1, which causes GSDMD cleavage detected by Western blot (Figure 2B, lower panel), abolished the luciferase activity of GSDMD-BS (Figure 2B, upper panel).

**Figure 3:**
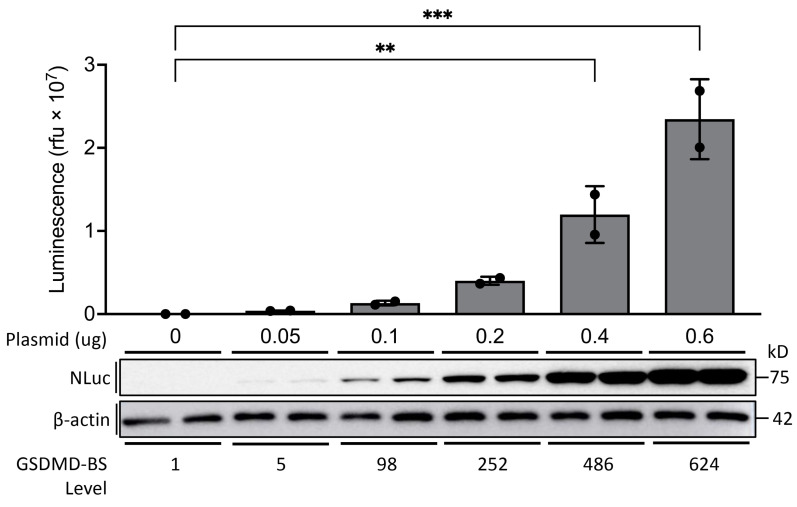
**Dose-dependent transfection of GSDMD-BS in HEK293T cells.** The GSDMD-BS/pBiT1.1-N construct was transfected in duplicate into HEK293T cells in a 12-well plate at increasing quantities (0, 0.05, 0.1, 0.2, 0.4, 0.6 μg). Protein lysates were extracted for luciferase assays and Western blot analysis. Upper panel: Luciferase signal increases in a dose-dependent manner to plasmid amount transfected, relative to the control (*n* = 2; One way ANOVA analysis **, *p* < 0.01; ***, *p* < 0.001). Lower panel: Protein expression detected in the Western blot increases at a consistent rate to the luciferase activity. GSDMD-BS protein levels in Western blot were quantified using ImageJ (normalized to β-actin internal control).

Next, we tested whether this GSDMD-BS can quantify the levels of GSDMD expression in cells. As shown in Figure 3, transfection of increasing amounts of plasmid expressing GSDMD-BS into HEK293T cells leads to increasing levels of GSDMD-BS detected by Western blot using anti-NanoLuc (NLuc) antibody (Figure 3, low panel), which is positively correlated with luciferase activity measured by luciferase assays (Figure 3, upper panel), demonstrating that this biosensor can quantify GSDMD levels in cells as anticipated (Figure 1C).

### 3.2. Bioluminescent imaging (BLI) Analysis of GSDMD-BS Activity In Vitro and In Vivo

**Figure 4:**
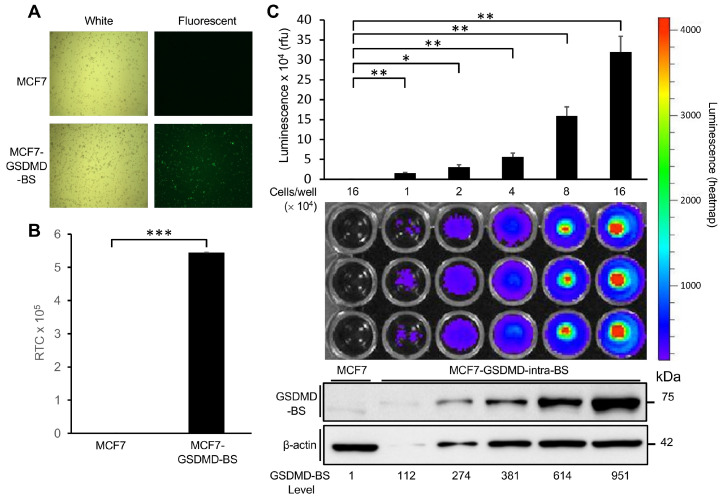
**Detection of GSDMD-BS by luciferase and bioluminescent imaging analysis.** GSDMD-negative MCF7 cells were stably infected with lentivirus expressing GFP and GSDMD-BS. (**A**) Images of MCF7 and MCF7-GSDMD-BS cells taken under white light (left) and blue light (right, green fluorescence), respectively. Pictures were taken with a Nikon TE-2000U microscope. (**B**) Luciferase assays. Proteins were extracted from MCF7 and MCF7-GSDMD-BS cells, followed by luciferase assays. High luminescence signal (***, *p* < 0.001) indicating GSDMD-BS expression (mean ± SD; *n* = 3). (**C**) Bioluminescent imaging analysis of GSDMD-BS. Increasing numbers of MCF7 cells stably expressing GSDMD-BS (1–16 × 10^4^) were seeded into a 96-well plate in triplicate, along with MCF7 cells as the control. Furimazine was added directly before imaging took place. A heatmap of signal counts represents luminescence. GSDMD-BS luminescence signal was clearly visualized using BLI analysis (middle panel)**,** then raw luminescence was measured using the NanoGlo Navigator (mean ± SD; *n* = 3; *, *p* < 0.05; **, *p* < 0.01)**.** GSDMD-BS levels detected by Western blot (lower panel) were consistent with luminescence data (upper panel). Protein levels were quantified using ImageJ (normalized to β-actin internal control).

To perform BLI analysis, we first established a GSDMD-negative MCF7 cell line stably expressing GSDMD-BS (MCF7-GSDMD-BS) (Figure 4A). Compared to MCF7, bioluminescent signals of MCF7-GSDMD-BS are very high in living cells (Figure 4B). Further BLI analysis of MCF7-GSDMD-BS shows that BLI signals (Figure 4C, middle panel) in living cells are positively correlated with levels of GSDMD-BS detected by Western blot analysis (Figure 4C, low panel) and luciferase activity measured in vitro using protein lysates extracted from equal numbers of cells used for BLI analysis (Figure 4C, upper panel).

**Figure 5:**
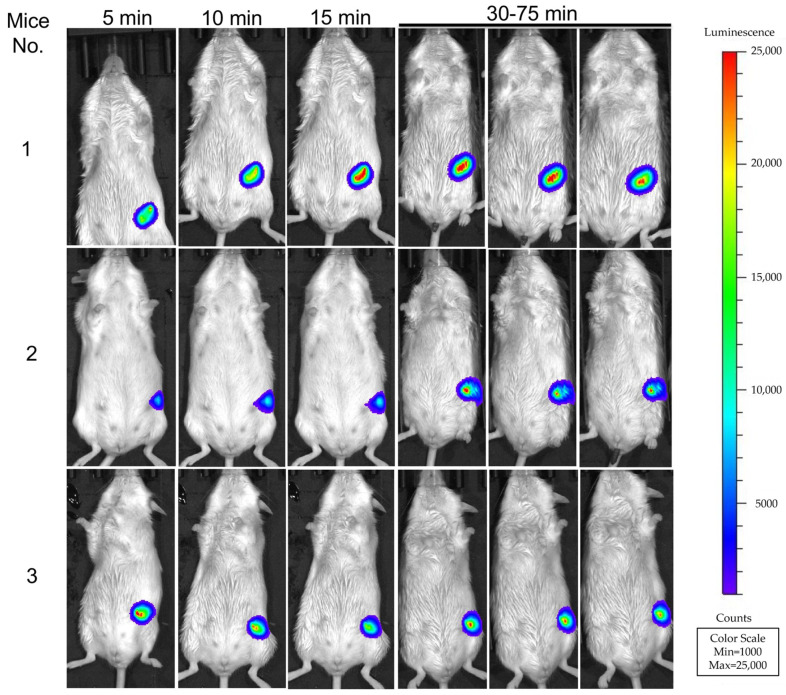
In vivo detection of GSDMD-BS activity in xenograft mice. BLI of 3 mice 5–75 min after flank implantation of fluorofurimazine preincubated cells. MCF7 control cells were implanted in the left flank, while MCF7-GSDMD-BS cells were implanted in the right flank of each mouse. The bioluminescent signal is only detectable in the right flank, indicating the presence and activity of the GSDMD-BS in the MCF7-GSDMD-BS cells. Quantification of bioluminescence is indicated on the right panel of this figure.

To detect GSDMD-BS in a xenograft mouse model in vivo, we injected MCF7 or MCF7-GSDMD-BS cells into the mammary pad of immunocompromised mice. Significantly, while no bioluminescent signals were detected after MCF7 injection (left flank of mice), strong signals were detected up to 75 min after MCF7-GSDMD-BS injection (right flank of mice, Figure 5), demonstrating that this GSDMD biosensor can represent GSDMD levels and intramolecular interactions in vivo.

### 3.3. Characterization of GSDMD-BS In Vitro

**Figure 6:**
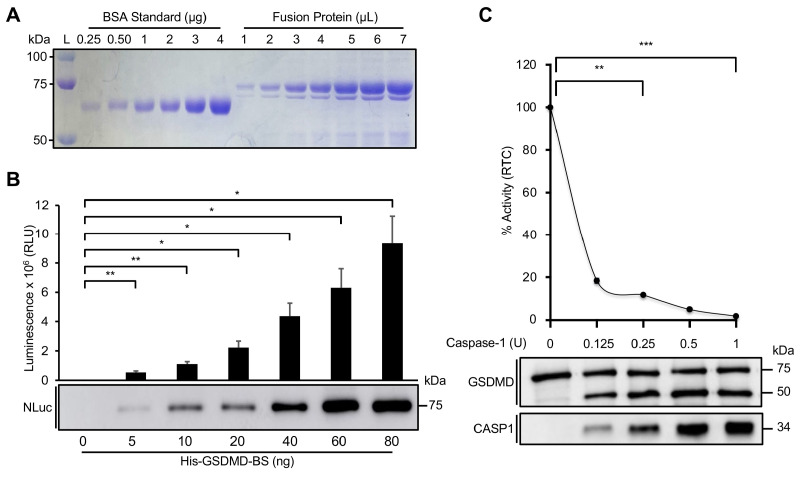
Characterizing GSDMD-BS activity in vitro. (**A**) Purification of His-GSDMD-BS fusion protein. The His-GSDMD-BS was purified and quantified using BSA as a standard (0.25–4 μg). Fusion protein is estimated to be approximately 0.25 μg/μL from the Coomassie blue staining. (**B**) Luciferase assay of His-GSDMD-BS in vitro. Increasing amounts (0–80 ng) of purified fusion protein were use for luciferase assays in vitro, demonstrating the biosensor’s high sensitivity at relatively low quantities (mean ± SD; *n* = 2), along with a steady increase in protein levels from the Western blot. *, *p* < 0.05; **, *p* < 0.01. (**C**) Caspase-mediated cleavage of GSDMD-BS in vitro. Purified CASP1 cleaves His-GSDMD-BS fusion protein (100 ng) at a high rate. Upper panel: Luminescent signal reduces drastically when His-GSDMD-BS is incubated with increasing amounts [0–1 Unit (U)] of CASP1 (mean ± SD; *n* = 2; **, *p* < 0.01; ***, *p* > 0.001). Lower panel: Increased cleaved GSDMD (50 kDa) is detected using anti-GSDMD antibody, along with a steady increase in CASP1 levels detected using anti-CASP1 antibody.

We further characterized GSDMD-BS in vitro using His-tagged GSDMD-BS fusion protein purified from bacteria (Figure 6A). Significantly, luciferase activity exhibited a positive correlation with the quantities of purified His-GSDMD-BS used in the assays (Figure 6B). In addition, GSDMD-BS luciferase activity shows a dose-dependent reduction with increased GSDMD cleavage caused by the addition of higher amounts of active caspase-1 in the assays (Figure 6C). In summary, this purified fusion protein of the GSDMD biosensor serves as an effective tool for accurate quantification of GSDMD’s levels, intramolecular interaction, and cleavage in vitro.

## 4. Discussion

The development and characterization of the GSDMD bioluminescent biosensor (GSDMD-BS) described in this study represents a significant advancement in our ability to monitor GSDMD dynamics in real-time, both in vitro and in vivo. This novel tool provides valuable insights into GSDMD’s intramolecular interactions, cleavage by caspases, and overall expression levels, which are critical for understanding its role in pyroptosis and related inflammatory processes.

Our results demonstrate that GSDMD-BS effectively reports on the intramolecular interaction of GSDMD. The dramatic increase in luminescent activity observed when GSDMD-BS is expressed, compared to controls, indicates successful complementation of the NanoBiT luciferase components LgBiT and SmBiT (Figure 1C,D). This was further validated by the near-complete loss of signal in the GSDMD-MUT construct, which contains mutations known to disrupt NTD-CTD binding (Figure 2A). These findings confirm that the biosensor’s signal is indeed dependent on GSDMD’s closed conformation, making it a reliable tool for studying GSDMD conformation and activation.

The GSDMD-BS also proved highly sensitive to caspase-mediated cleavage, as evidenced by the significant reduction in luminescent signal upon co-expression with active caspase-1 (Figure 2B). This mirrors the physiological activation of GSDMD during pyroptosis and demonstrates the biosensor’s potential for monitoring GSDMD cleavage in real-time. Such capability is particularly valuable for studying the kinetics of GSDMD activation in various cellular contexts and in response to different stimuli. In addition, the characterization of purified His-tagged GSDMD-BS fusion protein provides additional validation of the biosensor’s functionality and specificity. The dose-dependent responses observed with increasing protein quantities (Figure 6B) and caspase-1-mediated cleavage (Figure 6C) in cell-free systems underscore the biosensor’s potential for in vitro biochemical assays and drug screening applications.

Importantly, we have shown that the GSDMD-BS can quantitatively report on GSDMD expression levels in cells. The strong positive correlation between biosensor signal and protein levels detected by Western blot (Figure 3) indicates that the biosensor can serve as a non-invasive method for monitoring GSDMD abundance. This feature could be particularly useful for high-throughput screening of upstream regulators or drugs that modulate GSDMD stability. This finding is consistent with previous reports of chemotherapy-induced pyroptosis [1,2] and suggests that our biosensor could be a valuable tool for identifying and characterizing novel compounds that modulate GSDMD activity or expression.

The successful establishment of a stable cell line expressing GSDMD-BS (MCF7-GSDMD-BS) and its application in live cell bioluminescent imaging (BLI) demonstrates the versatility of this tool for both in vitro and in vivo studies. The strong correlation between BLI signals, Western blot analysis, and in vitro luciferase assays (Figure 4C) further validates the reliability of the biosensor across different experimental platforms. Moreover, the detection of GSDMD-BS activity in a xenograft mouse model (Figure 5) opens up exciting possibilities for studying GSDMD dynamics in complex physiological environments and disease models. It can also be used to test the effect of GSDMD inhibitors on GSDMD function in vivo.

While our current study focuses on GSDMD’s role in pyroptosis, the versatility of this biosensor design suggests potential applications in studying other pathways. For instance, the GSDMD-BS could potentially be adapted to investigate cross-talk between pyroptosis and other signaling pathways, such as the Hippo pathway [30], and the recently discovered link between GSDMD and metabolic regulation in tissue repair [14]. Future studies could explore the use of this biosensor design in mapping the complex network of interactions involving GSDMD in various cellular processes.

While our study presents a significant advancement in GSDMD research tools, it is important to acknowledge certain limitations. The biosensor’s reliance on the overexpression of a modified GSDMD protein may not fully recapitulate the behavior of endogenous GSDMD in all contexts. Additionally, the presence of large protein tags (LgBiT and SmBiT) could potentially affect GSDMD’s interactions with other proteins or its subcellular localization. Future studies should address these potential issues by comparing the biosensor’s readings with other established methods for measuring endogenous GSDMD activity and by developing knock-in models expressing the biosensor from the endogenous GSDMD locus.

## 5. Conclusions

In conclusion, the GSDMD bioluminescent biosensor described in this study represents a significant technological advance in the field of pyroptosis research. By enabling real-time, quantitative monitoring of GSDMD intramolecular interactions, cleavage, and expression levels in living cells and animal models, this tool has the potential to accelerate our understanding of GSDMD’s roles in health and disease. The versatility and sensitivity of the GSDMD-BS make it a valuable asset for both basic research and drug discovery efforts aimed at modulating pyroptosis and related inflammatory processes.

## Data Availability

No data can be shared.

## Supplementary Materials

The following supporting information can be downloaded at https://www.mdpi.com/article/10.3390/cells13191606/s1: Table S1: List of primers used for PCR.
