# Supplementary material for: A Novel Bioluminescent Biosensor Quantifying Intramolecular Interaction and Levels of Pyroptosis Effector GSDMD
Source: Cells. 2024 Sep 25;13(19):1606. doi: 10.3390/cells13191606 (PMC11475789; doi:10.3390/cells13191606)
Supplement: Supplementary file 1 [file cells-13-01606-s001.zip › cells-3220328-supplementary.pdf]

Table S1. List of Primers Used for PCR

Construct	Primer No.	Primer Name	Sequence (5′ to 3′)
GSDMD-BS (GSDMD-Sm/pBiT1.1-N)	1	EcoRI-NoStart-GSDMD-F	GGAATTCGGGGTCGGCCTTTGAGCGG
	2	BglII-Stop-SmBiT-linker-NoStop-GSDMD-R	GAAGATCTCTACAGAATCTCCTCGAACAGCCGGTAGCCGGTCACACCTGACGACCCTCCACCTCCGCTCCCGCCACCACCGTGGGGCTCCTGGCTCAG
GSDMD(MUT)-BS (GSDMD(MUT)-Sm/pBiT1.1-N)	3	EcoRI-NoStart-GSDMD-F	GGAATTCGGGGTCGGCCTTTGAGCGG
	4	BglII-Stop-SmBiT-linker-NoStop-GSDMD-R	GAAGATCTCTACAGAATCTCCTCGAACAGCCGGTAGCCGGTCACACCTGACGACCCTCCACCTCCGCTCCCGCCACCACCGTGGGGCTCCTGGCTCAG
	5	GSDMD-L290A-E293A-F	GAAGACTTCCAGGGCGCACGGGCAGCGGTGGAGACCATCTCC
	6	GSDMD-L290A-E293A-R	GGAGATGGTCTCCACCGCTGCCCGTGCGCCCTGGAAGTCTTC
	7	GSDMD-Y373A-A377D-F	GAACTCGCTATCCCTGTTGTCGCCCTGCTGGGGGACCTGACCATGCTGAGTGAAACG
	8	GSDMD-Y373A-A377D-R	CGTTTCACTCAGCATGGTCAGGTCCCCCCAGCAGGGCGACAACAGGGATAGCGAGTTC
	9	GSDMD-A471D-F	CATGTGTGCACTCTACGACTCCCTGGCACTGCTATC
	10	GSDMD-A471D-R	GATAGCAGTGCCAGGGAGTCGTAGAGTGCACACATG
GSDMD-BS (stable line) (Lg-GSDMD-Sm/WPI-HA)	11	BamHI-Start-LgBiT-F	GCAGGATCCATGGTCTTCACACTCGAAGATTTCGTTGGG
	12	MLu1-Stop-SmBiT-R	CCGACGCGTTTAGAGAATCTCCTCGAACAGCCG
FLAG-CASP1(cat) (CASP1(cat)/pcDNA3.1-3xFLAG-hygro)	13	BamH1-CASP1-120F	CTGGATCCAACCCAGCTATGCCCACATCC
	14	Not1-Stop-CASP1-404R	ATGAAACTGCGGCCGCTTAATGTCCTGGGAAGAGGTAGAAAC
